# Impact of the left anterior descending artery wrapping around the left ventricular apex on cardiac mechanics in patients with normal coronary angiography

**DOI:** 10.1186/s43044-020-00059-z

**Published:** 2020-06-03

**Authors:** Hala Mahfouz Badran, Waleed Abdou Ibrahim, Tamer Alaksher, Ghada Soltan

**Affiliations:** 1 Menoufia University, P.O box 34, 55-El Gish street, Tanta, Egypt; 2 National Heart Institute, Cairo, Egypt

**Keywords:** Left anterior descending wrapping, LV mechanics, Speckle tracking

## Abstract

**Background:**

We examined the impact of left anterior descending (LAD) wrapping on left ventricular (LV) mechanics in patients with normal coronary angiography. Seventy-one patients with evidence of normal coronary angiography (LAD wrapping: *n* = 52, 73%) and LAD non-wrapping (*n* = 19, 27%) were included in the study. Using 2D-strain imaging, we measured LV longitudinal and circumferential (circ) strain (ε_sys_), systolic strain rate (SR_sys_), early (SR_e_) and atrial (SR_a_) diastolic SR, LV electromechanical dyssynchrony (TTP-SD), and LV twist and torsion in study groups.

**Results:**

No significant difference in age, gender, body surface area (BSA), or ejection fraction (EF%) between groups. LAD-wrapping group showed higher deceleration time (DT) (*P* < 0.0001), global longitudinal ε_sys_ % (*P* < 0.02), circ SR_a_ at the basal segments (*P* < .02), circ SR_sys_ and SR_e,_ and SR_a_ (*P* < 0.0001) at the apical segments and apical rotation compared with the non-wrapped group. LV twist was correlated negatively with LV electromechanical dyssynchrony (*r* = .25, *P* < 0.03) and positively with longitudinal ε_sys_ (*r* = .47, *P* < .0001), circ ε_sys%_ (*r* = .55, *P* < .0001), circ SR_sys_ (*r* = .23, *P* < .05), and circ SR_e_ (*r* = .55, *P* < .0001). Using multivariate regression analysis, DT: OR 0.932, CI 0.877–0.991, and *P* < 0.02 and circ at atrial diastole (SR_a_): OR 0.000, CI .000–.271, and *P* < 0.03 were independent predictors of LAD wrapping around LV apex.

**Conclusion:**

Wrapped LAD is associated with better myocardial relaxation and rotational mechanics in patients with normal coronary angiography. This could explain the worse prognosis in such population when LAD occlusion acutely emerges.

## Background

Nowadays, coronary artery disease represents 31% of deaths and the most important cause of morbidity and mortality all over the world [1]. The left anterior descending coronary artery (LAD) supplies blood flow to the greater part of the cardiac muscle. Nevertheless, the amount of myocardium supplied with LAD differs according to its length, and consequently, the site of coronary artery occlusion could influence the patient outcome.

Earlier studies [2, 3] described the LAD anatomical course, and divisions are varied in approximately 78% of healthy individuals. The LAD route may extend around the apex and terminates beyond the diaphragmatic aspect of the left ventricle (LV). Nonetheless, LAD fails to continue on the diaphragmatic surface in 22% of patients; alternatively, it terminates at or even before the cardiac apex [4]. Further research work described the LAD coronary artery to be wrapped around the cardiac apex in 47% of individuals with right coronary artery dominance and in 87% of those with left coronary dominance [3].

Myocardial blood flow through LAD constitutes the largest cardiac blood supply that explains the poor patient’s prognosis when proximal LAD occlusion developed. Proximal LAD occlusion would jeopardize a large portion of the myocardium and render the patient at a greater risk of heart failure and cardiac death [5–8]. The association between poor patients outcomes in anterior ST-segment elevation myocardial infarction (STEMI) and LAD anatomic features was lately explored [9, 10]. During long-term follow-up, several investigators clarified the impact of LAD wrapping around the cardiac apex and the occurrence of adverse cardiovascular events due to the larger area of infarct [9, 10].

However, there is a lack of records in the literature as regards the relationship of LAD anatomical features and LV mechanics in patients with normal coronary angiography.

Two-dimensional (2D) strain imaging has been introduced as a novel method with angle-independency, acceptable reproducibility, and segmental quantification of LV strain (ε_sys_), strain rate (SR), and LV twist [11–14]; by these measurements, LV function could be assessed.

The aim of the present study was to examine functional characteristics of cardiac mechanics using two-dimensional strain imaging (2D-strain) in patients with and without LAD wrapping around left ventricular apex in angiographically normal coronary arteries.

## Methods

### Study population

This is cross-sectional study included all patients who were scheduled for elective coronary angiography for the first time at our catheterization laboratory (university hospital) and have normal coronary angiography through the period from January 2016 to December 2018.

### Inclusion criteria

Patients aged 18 years or more who were scheduled for elective coronary angiography and have normal coronary angiography presented with chest pain or referred for evaluation of coronary arteries to rule out coronary artery disease (CAD) were enrolled in this study.

### The exclusion criteria

Patients with evidence of obstructive CAD, previous acute coronary syndrome, prior coronary intervention/coronary artery bypass graft surgery, anomalies of coronary arteries, moderate-to-severe valvular regurgitation or any valvular stenosis, heart failure, reduced ejection fraction (EF), left bundle branch block ( LBBB), atrial fibrillation, or malignancy were all excluded from the study. Written informed consent was obtained from the participants. The study proposal was approved by the Ethics Committee and the Research Board of the University. Patients were recruited and examined in Yacoub Research Unit, in our university.

### Coronary angiogram

All patients were subjected to elective coronary angiography using standard procedures and multiple projections and angles. Coronary arteries were assessed according to LAD termination character. “LAD wrapping” was defined as an LAD artery that perfuses at least one fourth of the LV inferior wall in the right anterior oblique and caudal projection, and it does not terminate at the apex but extends through the diaphragmatic surface. While LAD non-wrapping was defined when LAD does not expand on the diaphragmatic surface and terminates at the apex 1–4]. Angiographic data was reviewed by at least two experienced observers.

A normal coronary angiogram was defined as one with the absence of any identifiable pathology (comprises signs of atherosclerosis, spontaneous coronary spasm, or thrombosis) [1]. Entirely, patients had mild coronary artery atherosclerotic lesions with ≤ 50% diameter stenosis, were diagnosed as having an obstructive coronary disease and excluded from the study.

From 1600 patients who undergone elective angiography, 71 meet our inclusion criteria. The patients were categorized into two groups according to LAD anatomic feature: those with a long LAD (wrapping around LV apex) constituted (LAD wrapping group, *n* = 52) and those with the LAD terminating at or before the apex constituted (LAD non-wrapping group, *n* = 19).

### Conventional echocardiography

All included patients underwent an echocardiographic examination within 1 week of coronary angiographic study using an ultrasonographic system (Esaote Mylab Gold 30 ultrasound system (Esaote S.p.A, Florence, Italy) equipped with a multi-frequency 2.5–3.5 MHz phased-array transducer was employed. LV end-diastolic and systolic (EDD, ESD) diameters, septum (SPT) and posterior (PWT) wall thickness, LV mass (LVM), and LVM index (LVMI) were calculated in accordance with the recommendations of the American Society of Echocardiography [15]. LV EF% was measured using a modified Simpson’s rule. Left atrial (LA) diameter and volume (LAV) were calculated using the biplane area-length method and indexed for BSA [15]. Measurements of pulmonary artery pressure (PAP) were carried out using tricuspid regurgitation velocity-derived Bernoulli equation (PG = 4 V2).

Mitral inflow velocity patterns were recorded from apical 4CH view (4–5 mm) sample volume width was utilized to be placed at the mitral leaflet tips, in diastole and using 3 consecutive cardiac cycles. An optimized pulsed wave Doppler beam parallel to the direction of mitral inflow was taken. Peak early and late diastolic trans-mitral velocities (E&A) and E-wave deceleration time were measured. All pulsed Doppler signals were recorded at a horizontal sweep of 100 mm/s [15].

LV diastolic function assessment was performed using conventional transmitral flow velocity-derived indices. Additionally, the mitral annular motion was recorded from the apical 4CH view using a tissue Doppler imaging program (TDI) [16]. A 4–5-mm sample volume was placed sequentially at the lateral and then septal corners of the mitral annulus. Peak systolic (S') early diastolic (E') and late diastolic annular velocities were measured. Annular velocities were recorded during end-expiratory phase, at sweep speed 50 to 100 mm/s and averaged from 3 consecutive cardiac cycles at both annuli [17]. E/E’ as a measure of LV end-diastolic pressure was calculated [15, 17].

### Analysis of LV deformation

Both LV segmental and global mechanics were analyzed using vector velocity imaging (VVI). It is an angle-independent feature-tracking mode that encompasses speckle tracking with endocardial and epicardial contour tracking. It measures myocardial motion from B-mode clips by automatically tracing contours to define the inward and outward myocardial motion [18].

Strain and SR measurements were recorded from apical 2CH and 4CH views at the frame rate (90 ± 20 F/s) according to the heart rate. The images and clips were stored, in digital format for subsequent offline analysis. Complete breath-holding during expiration was ensured to avoid excessive translational motion. Tracking and consequent strain analysis were performed using Esaote-X-Strain software package according to the validated algorithm [18]. Peak longitudinal systolic ε_sys_, systolic SR (SR_sys_), early (SR_e_), and atrial (SR_a_) diastolic SR of the basal, mid, and apical segments at the mid myocardial layer of septal, lateral, anterior, and inferior walls were measured and averaged to calculate the global deformation. Circ εsys was measured from short-axis views at the mid papillary levels, midway between the mitral valve and the apex; LV twist was measured from short-axis views at the basal and apical levels; the basal short-axis plane contained the mitral valve; and the apical plane was acquired distally to the papillary muscle, as circular as possible and proximal to the plane with luminal obliteration at end-systole. LV twist was calculated as the instantaneous difference between apical and basal rotation [18, 19].

LV mechanical dyssynchrony, from regional strain curves the time to peak strain (TTP), was estimated for each ventricular segment, as the time from the beginning of the QRS complex of the ECG to the peak ε_sys_. The electromechanical delay was measured as the difference between the longest and shortest TTP of the twelve LV myocardial segments [20]. LV dyssynchrony was defined as the standard deviation of the averaged TTP from all myocardial segments (TTP-SD) [20].

## Statistical analysis

Values were presented as means ± SD or as numbers and proportions, as appropriate. The relations between qualitative variables were evaluated by chi-square test or Fisher’s exact test, as indicated. Means were compared with Student’s *t* test. Pearson correlation coefficient analysis was used to test the relation of LV twist and all clinical and echocardiographic variables. Variables that were significantly associated with patient’s groups (wrapping versus non-wrapping) on univariate analysis were introduced into the multivariate logistic regression model to detect independent predictors. The analysis was performed by statistical package software IBM- SPSS for MAC, version 25. All tests were bilateral and a *P* value of < 0.05 was considered statistically significant.

## Results

In total, 71 patients having angiographically normal coronary arteries were included in the study; those with a long LAD wraparound LV apex constituted 73% (LAD wrapping group, *n* = 52) and those with the LAD terminating at or before LV apex constituted 27% (LAD non-wrapping group, *n* = 19).

### Patients’ characteristics

Patient’s clinical characteristics were depicted in Table 1. No significant difference between study groups in age between LAD wrapping group 52 **±** 7.18 and LAD non-wrapping group 50.4 **±** 8.7, *P* = NS; the male to female ratio was 1.53 [31 (59.6%) males and 21 (40.4%) females], among LAD wrapping group, and 1.36 [11 (57.9%) males and 8 (42.1 %) females]. Patients with LAD wrapping have a higher prevalence of hypertension (62% versus 26%, *P* < .008), lower prevalence of diabetes mellitus (59.6% versus 100%, *P* < .001), and less prevalence of cigarette smoking (16% versus 46%, *P* < .000) compared with LAD non-wrapping. No differences between patients groups in BSA, heart rate, blood pressure, or family history of CAD.

**Table 1:** Clinical and echocardiographic findings in studied groups

	LAD wrapping (***n*** = 52)	LAD non-wrapping (***n*** = 19)	***P*** value		LAD wrapping (***n*** = 52)	LAD non-wrapping (***n*** = 19)	***P*** value
**Age (year)**	52.13 ± 7.18	50.42 ± 8.67	0.40	**Apical Lat**	− 13.21 ± 5.3	− 13.01 ± 2.5	0.874
**BSA (m**^**2**^**)**	1.99 ± 0.13	1.93 ± 0.13	0.084	**Mean Lat**	− 15.09 ± 6.1	− 15.29 ± 3.8	0.892
**Hieght (cm)**	172.2 ± 14	167.7 ± 19	0.300	**Basalinferior**	− 15.28 ± 4.9	− 15.79 ± 6.2	0.725.
**Weight (kg)**	89.2 ± 15.2	88.11 ± 25.3	0.997	**Mid inferior**	− 13.76 ± 5.8	− 14.49 ± 5.1	0.634
**BMI (kg/m**^**2**^**)**	1.72 ± 0.15	1.67 ± 0.19	0.542	**Apical inf**	− 13.75 ± 4.2	− 13.44 ± 3.9	0.779
**Hypertension**	32(61.5%)	5(26.3%)	.008	**Mean inf**	− 14.27 ± 4.5	− 14.57 ± 4.6	0.803
**Diabetes mellitus**	31(59.6%)	19(100%)	.001	**Basal ant**	− 17.32 ± 6.9	− 15.9 ± 3.9	0.420
**Smoking**	4(16.7%)	6(46.2%)	.000	**Mid ant**	− 14.62 ± 7.4	− 15.04 ± 2.4	0.785
**FH of CAD**	13(25%)	4(21.1%)	.730	**Apical ant**	− 13.77 ± 3.8	− 14.73 ± 2.2	0.295
**SBP (mmHg)**	132 ± 11	132.4 ± 12.1	0.961	**Mean ant**	− 15.23 ± 5.0	− 15.26 ± 2.6	0.985
**DBP (mmHg)**	78.75 ± 7.85	75.79 ± 6.72	0.149	**Global**	− 14.80 ± 3.6	− 14.9 ± 2.7	0.836
**HR (b/min)**	78.63 ± 10.97	81.39 ± 6.31	0.318	**TTP (ms)**	348.41 ± 53	339.1 ± 32	0.482
**LAD (mm)**	37.06 ± 3.58	35.31 ± 3.36	0.73	**TTP-SD (ms)**	40.68 ± 33.12	30.06 ± 32.5	0.204
**LA V(ml/m**^**2**^**)**	52.52 ± 15.77	45.94 ± 9.72	0.102	**TTP-d (ms)**	131.3 ± 91	98.0 ± 64.7	0.161
**ESD (mm))**	29.96 ± 3.75	28.61 ± 3.47	0.184	**Longitudinal SR**_**sys**_**(s**^**-1**^**)**			
**EDD (mm)**	47.4 ± 4.7	45.0 ± 5.3	0.074	**Septum**	− 1.91 ± 7.34	− 0.84 ± 0.42	0.53
**Septum (mm)**	10.29 ± 2.0	9.70 ± 2.4	0.344	**Lateral**	− 0.92 ± 0.32	− 0.91 ± 0.28	0.79
**PW (mm)**	10.28 ± 1.9	9.60 ± 2.4	0.213	**Anterior**	− 0.92 ± 0.31	− 1.06 ± 1.36	0.50
**FS%**	36.7 ± 4.9	37.2 ± 5.1	0.704	**Inferior**	− 0.83 ± 0.34	− 0.77 ± 0.19	0.46
**EF%**	65.1 ± 9.91	67.2 ± 5.66	0.405	**Global**	− 1.17 ± 2.02	− 0.89 ± 0.36	0.56
**LVM (gm)**	235.7 ± 55.8	193.1 ± 34.3	0.003	**Longitudinal SR**_**e**_**(s**^**-1**^**)**			
**LVMI (gm/m**^**2**^**)**	115.5 ± 28	92.9 ± 29	0.004	Mean septum	1.01 ± 0.40	0.92 ± 0.21	0.345
**Mitral E (cm/s)**	41.9 ± 21.5	50.3 ± 12.9	0.117	Mean lateral	1.03 ± 0.47	1 ± 0.27	0.819
**Mitral A (cm/s)**	53.1 ± 28	59.9 ± 11	0.325	Mean anterior	1.01 ± 0.46	0.92 ± 0.29	0.395
**Mitral E/A**	78.9 ± 16.9	0.84 ± 0.19	0.417	Mean inferior	1.00 ± 0.38	0.90 ± 0.26	0.257
**DT (msec)**	172.2 ± 38.4	131.9 ± 29.8	0.000	Global	0.72 ± 0.61	0.39 ± 0.30	0.026
**E/Ea**	4.68 ± 2.69	5.36 ± 1.7	0.302	**Longitudinal SR**_**a**_**(s**^**-1**^**)**			
**Longitudinal ε**_**sys**_**%**				Apical septum	0.54 ± 0.25	0.42 ± 0.14	0.052
**Basal septum**	− 15.10 ± 3.1	− 15.7 ± 3.7	0.561	Septum	0.85 ± 1.72	0.50 ± 0.42	0.374
**Mid septum**	− 14.67 ± 5.4	− 15.1 ± 3.1	0.717	Apical Lateral	0.55 ± 0.23	0.39 ± 0.11	0.005
**Apical septum**	− 13.59 ± 3.6	− 13.2 ± 3.1	0.679	Lateral	0.57 ± 0.21	0.45 ± 0.16	0.026
**Mean septum**	− 14.60 ± 3.8	− 14.9 ± 2.4	0.798	Apicalanterior	0.53 ± 0.24	0.36 ± 13	0.004
**basal lateral**	− 16.17 ± 8.6	− 17.0 ± 5.9	0.697	Anterior	0.61 ± 0.26	0.16 ± 1.07	0.006
**Mid lateral**	− 15.11 ± 5.9	− 14.9 ± 3.8	0.880	Global	0.72 ± 0.61	0.39 ± 0.30	0.026

*BSA* body surface area, *BMI* body mass index systolic blood pressure, *DBP* diastolic blood pressure, *LAD* left atrium dimension, *LAV* left atrium volume, *ESD* left ventricular end-systolic diameter, *EDD* left ventricular end-diastolic diameter, *EF* ejection fraction, *PW* posterior wall, *LVMI* left ventricular mass index, *E/A* ratio of early to late diastolic mitral inflow velocity, *DT* deceleration time, *E/Ea* ratio of early diastolic mitral inflow velocity to early diastolic mitral annular velocity, *ɛ*_*sys*_ peak systolic strain, *SR*_*sys*_ peak systolic strain rate, *SR*_*e*_ early diastolic strain rate, *SR*_*a*_ late diastolic strain rate

### Conventional echocardiographic findings

There was no significant difference between the two groups in LV dimensions, LA dimension, EF%, or E/E' as demonstrated in Table 1. Meanwhile, LVM, LVMI, and DT were significantly higher in LAD wrapping compared with LAD non-wrapping group; LVM: 235.7 ± 55.8 versus 193.3 ± 34, *P* < 0.003; LVMI: 115.5 ± 28 versus 92.9 ± 29, *P* < 0.004; and DT: 173.2 ± 38.3 versus 131.9 ± 29.8, *P* < 0.0001.

### Longitudinal strain

**Fig. 1:**
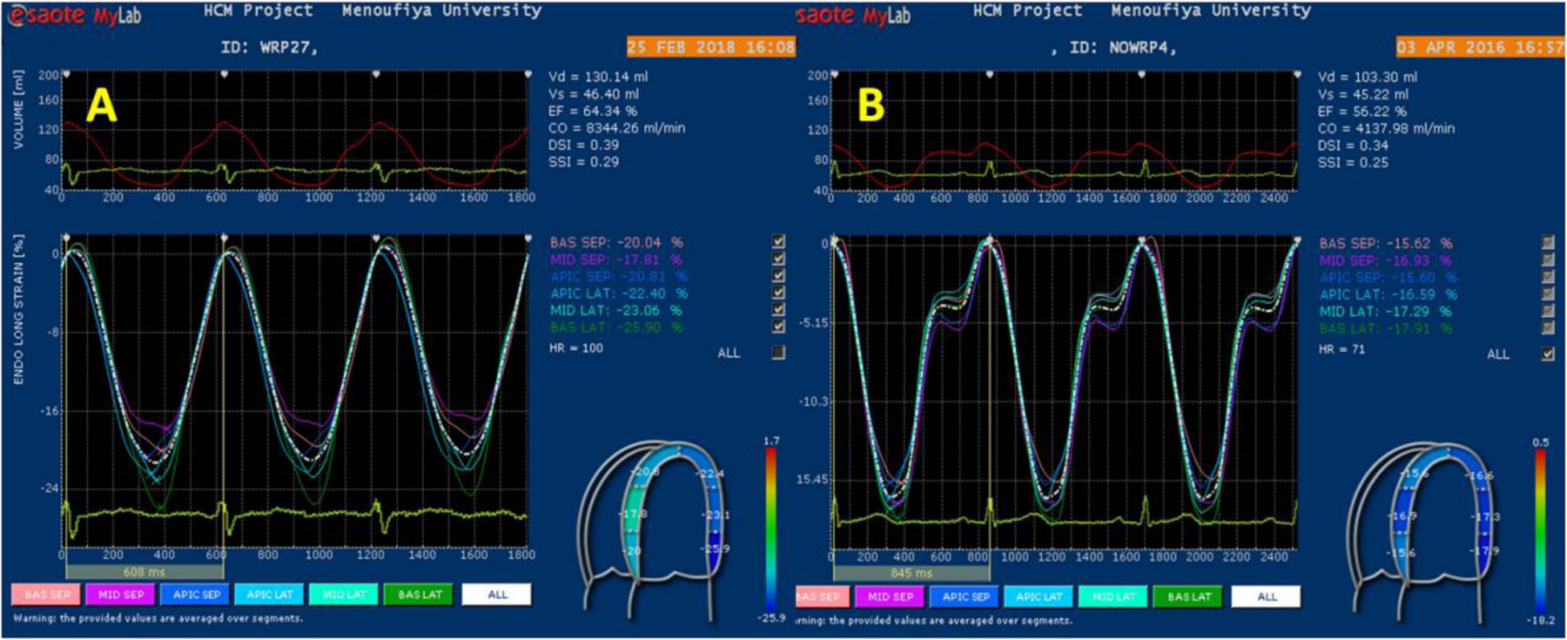
Longitudinal strain (LS) curves derived from LV segments in apical 4CH view. **a** LAD wrapping patient with mean LS = 21% and **b** LAD non-wrapping with mean LS = 17%

There was no significant difference between LAD wrapping and LAD non-wrapping groups in segmental or global longitudinal strain (ε_sys_) or systolic strain rate (SR_sys_) as demonstrated in Table 1. Meanwhile, no significant difference between the two groups in electromechanical delay or mechanical dyssynchrony (TTP-d, TTP-SD) (Fig. 1).

In the LAD wrapping group, SR_a_ of the apical septum (*P* < .05), apical lateral (*P* < .005), apical anterior (*P* < .004), lateral, and inferior was significantly greater than non-wrapping group (*P* < .001). Consequently, the global atrial diastolic strain rate (SR_a_) was significantly higher in the LAD wrapping group compared with LAD non-wrapping group *P* < 0.02 (Table 1).

### Circumferential strain

**Fig. 2:**
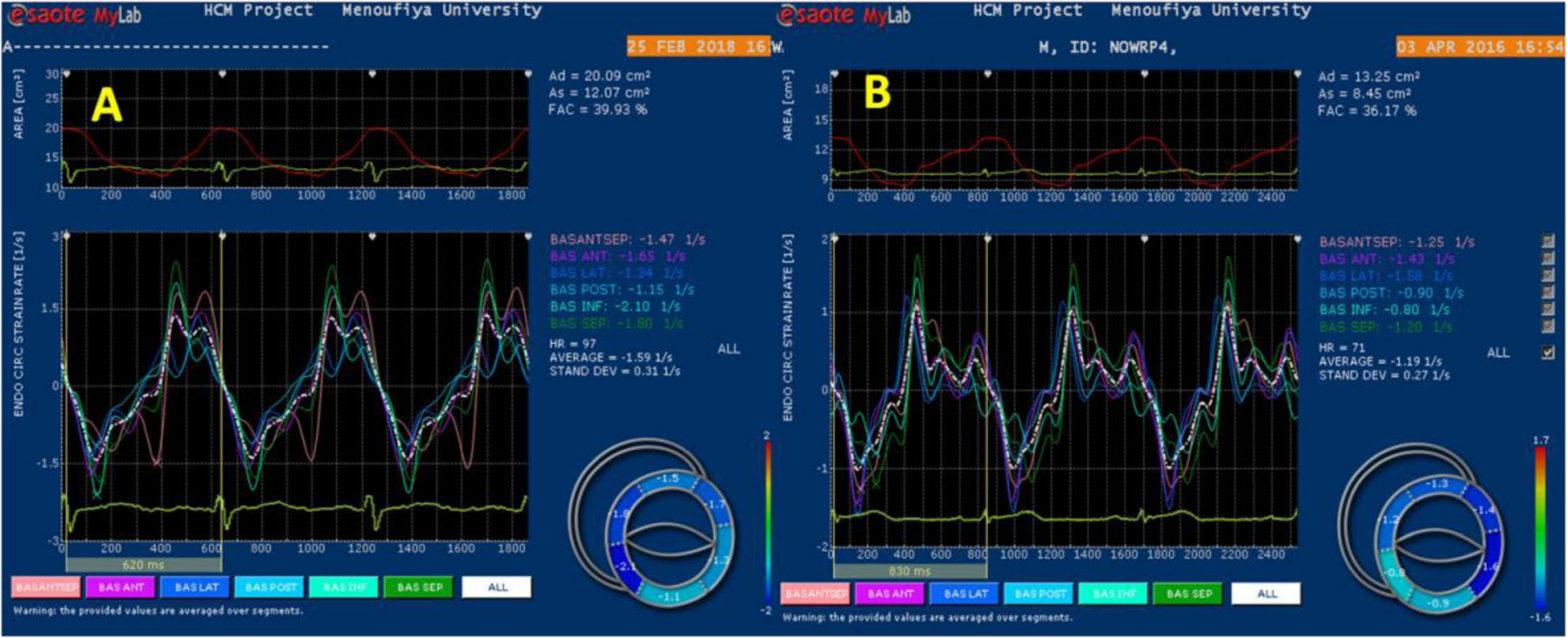
LV circumferential strain rate curves derived from short axis view at mitral valve annulus. **a** LAD wrapping patient with mean basal Circ **SR**_**a**_**1.23 (s-**^**1**^**)** and **b** LAD non-wrapping with mean basal Circ **SR**_**a**_**0.43 (s-**^**1**^**)**

**Fig. 3:**
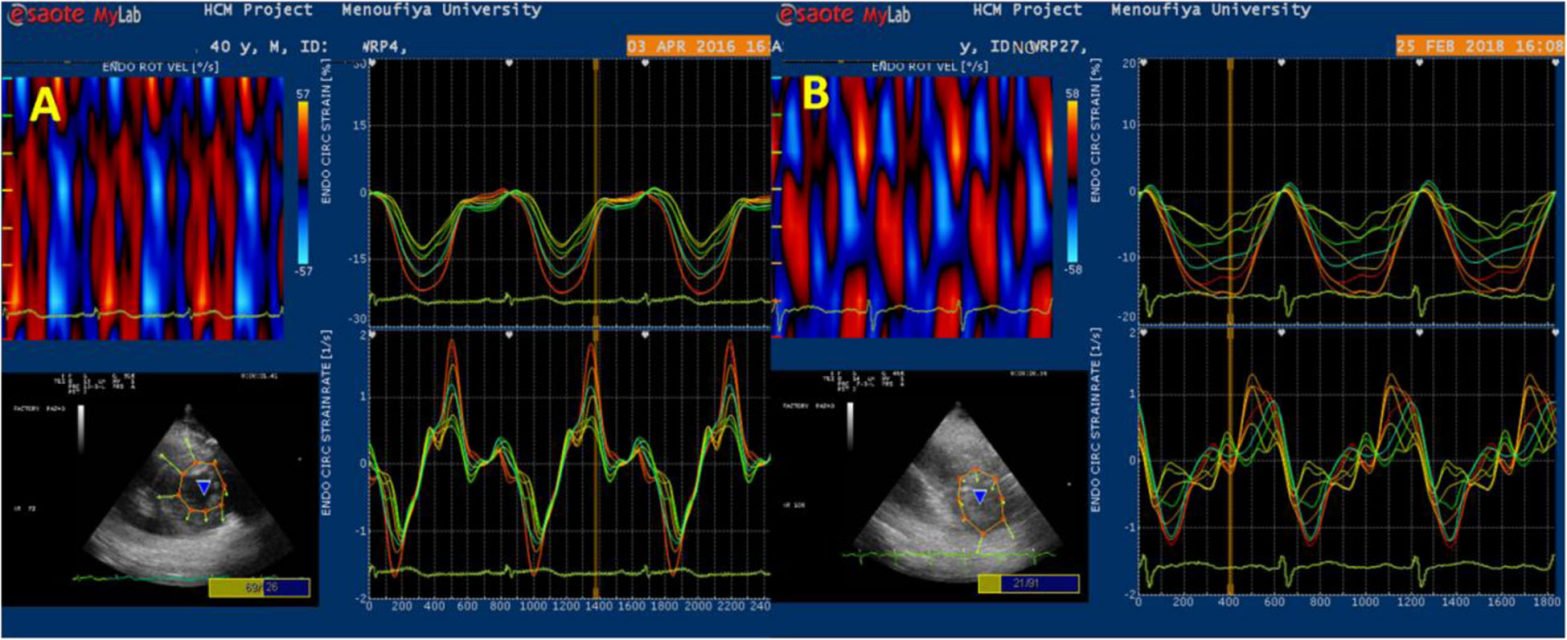
Circumferential strain and strain rate curves using VVI at short-axis view of LV apex. Upper left: curved M-mode of apical circ SR. Upper right: circ strain and lower right: circ SR curves. **a** LAD wrapping (mean value of circ ε_sys_ = 22%). **b** LAD non-wrapping (mean value circ ε_sys_ = 13%)

**Fig. 4:**
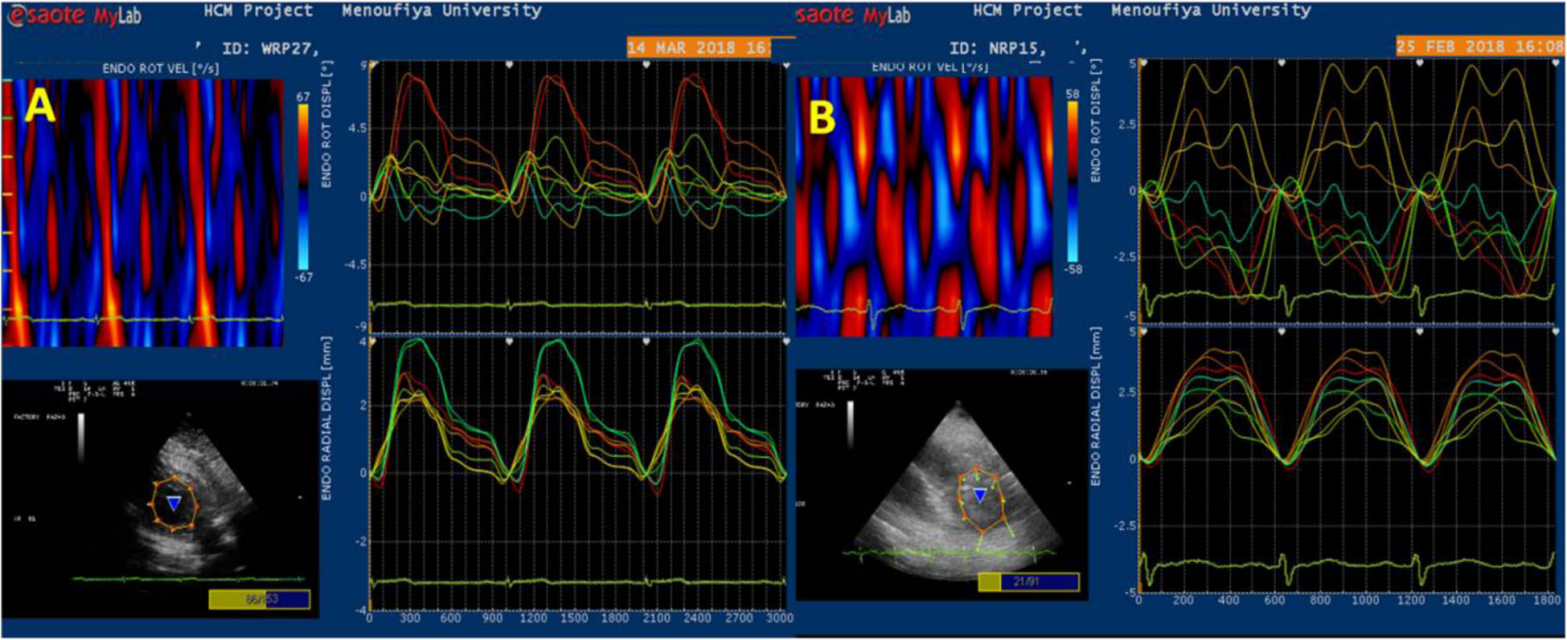
Apical rotational mechanics: upper left: curved M-mode of rotation velocity (°/s). Upper right: rotation degree of LV apex in **a** LAD wrapping (mean value = 5.5°). **b** LAD non-wrapping (mean value = 3.5°)

Circumferential ε_sys_% and SR were assessed at LV basal and apical planes in short-axis views (Tables 2 and 3). At basal level, LAD wrapping group showed higher circumferential SR_a_ during late diastole at segmental level; lateral (.54 ± .23 vs.38 ± .13, *P* < .005), posterior (.50 ± .21 vs.39 ± .18, *P* < .03), inferior (.53 ± .19 vs .36 ± .09, *P* < .0001), septum (.49 ± .15 vs .38 ± .082, *P* < .005), and global levels .74 ± 1.14 vs .38 ± .08, *P* < .01. However, there was no significant difference in circumferential strain or SR during systole or early diastole (Figs. 2, 3 and 4).

**Table 2:** Circumferential strain and strain rate at LV basal and apical segments

LV basal	LAD wrapping	LAD non-wrapping	***P*** value	LV apex	LAD wrapping	LAD non-wrapping	***P*** value
**Circ ε**_**sys**_**%**							
Mean basal	− 13.87 ± 4.0	− 13.5 ± 3.2	0.721	Mean apical	− 15.92 ± 4.5	− 13.78 ± 0.3	0.02
**Circ SR**_**sys**_**(s**^**-1**^**)**							
Mean basal	− 1.14 ± 1.83	−0 .87 ± .19	0.533	Mean apical	− 1.25 ± 1.85	− 0.84 ± .13	0.000
**Circ SR**_**e**_**(s**^**-1**^**)**							
Mean basal	0.96 ± .44	0.90 ± .25	0.564	Mean apical	1.09 ± 0.49	0.88 ± .18	0.000
**Circ SR**_**a**_**(s-**^**1**^**)**							
Basal septum	0.49 ± .15	0.38 ± .082	0.005	Apical anterior	0.56 ± .27	0.44 ± .14	0.053
Basal anterior	0.52 ± .25	0.41 ± .19	0.067	Apico septal	0.58 ± .30	0.37 ± .10	0.005
Basal lateral	0.54 ± .23	0.38 ± .13	0.005	Apical lateral	0.5635 ± .29	0.39 ± .10	0.017
Basal inferior	0.53 ± .19	0.36 ± .09	0.000	Apical infer	0.64 ± .43	0.36 ± .073	0.007
Mean basal	0.74 ± 1.14	0.38 ± .08	0.0121	Mean apical	0.58 ± .52	0.39 ± .062	0.000

*ɛ*_*sys*_ peak systolic strain, *SR*_*sys*_ peak systolic strain rat, *SR*_*e*_ early diastolic strain rate, *SR*_*a*_ late diastolic strain rate

**Table 3:** LV rotation in studied groups

	LAD wrapping (***n*** = 52)	LAD non-wrapping (***n*** = 19)	***P*** value
ROT basal anteroseptal	− 2.65 ± 2.25	− 2.22 ± 1.64	.453
ROT basal anterior	− 3.42 ± 2.34	− 2.91 ± 1.98	.404
ROT basal lateral	− 4.22 ± 1.96	− 3.39 ± 2.90	.174
ROT basal inferior	− 3.81 ± 1.81	− 3.92 ± 2.66	.843
ROT basal posterior	− 3.88 ± 2.45	− 4.44 ± 2.01	.374
ROT basal septum	− 3.82 ± 2.58	− 3.64 ± 1.78	.781
Mean basal ROT	− 4.80 ± 2.91	− 3.41 ± 2.12	.408
ROT apical septum	3.58 ± 1.31	2.69 ± 1.56	.019
ROT apical anterior	3.62 ± 1.32	2.57 ± 1.10	.003
ROT apical lateral	3.51 ± 1.78	2.54 ± 1.50	.087
ROT apical inferior	4.52 ± 1.79	3.70 ± 1.89	.218
Mean apical ROT	4.39 ± 1.28	3.03 ± 1.37	.003
Long axis	70.37 ± 13.83	73.87 ± 15.57	.518
Twist	8.91 ± 3.19	6.44 ± 2.09	.001
Torsion	0.12 ± 0.66	0.08 ± 0.71	.001

The main finding of circumferential strain analysis *at LV apex* was the higher values of circumferential ε_sys_%, SR_sys_, SR_e_, and SR_a_ in LAD wrapping group compared with LAD non-wrapping group; ε_sys_%: − 15.92 ± 4.46 versus − 13.78 ± 0.34, *P* < .02; SR_sys_: − 1.25 ± 1.85 versus − 0.84 ± 0.13, *P* < .0001; SR_e_: 1.09 ± 0.49 versus 0.88 ± 0.18, *P* < .0001; and SR_a_: .58 ± .52 versus .39 ± .062, *P* < .0001.

**Fig. 5:**
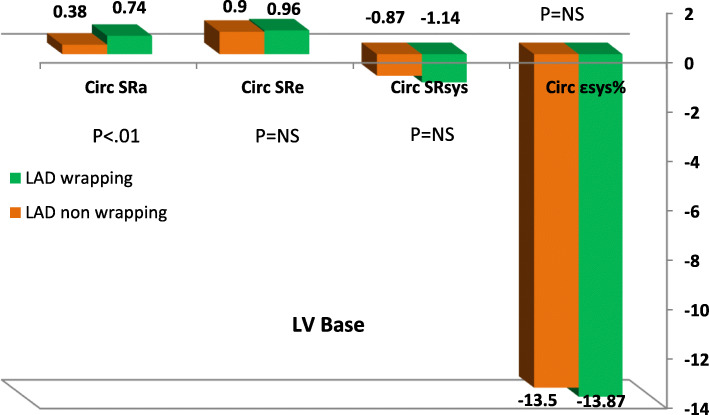
Circumferential strain and strain rate at LV basal segments

**Fig. 6:**
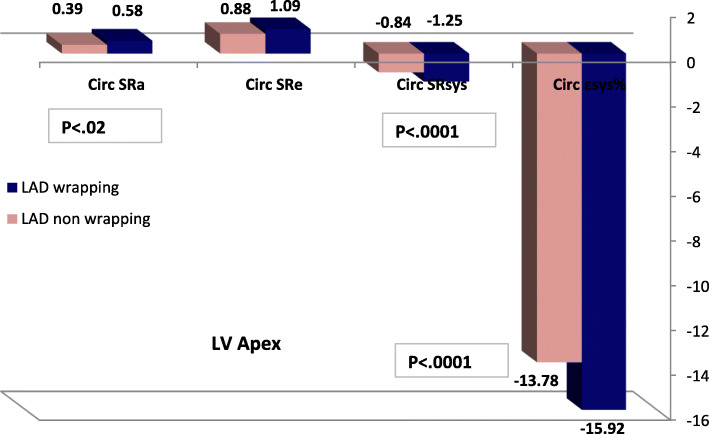
Circumferential strain and strain rate at LV apex in study groups

In the LAD wrapping group, there was a significant increase in apical septal (*P* < .01), apical anterior rotation with *P* < .003, and the averaged degree of LV apical rotation (*P* < .003) compared with LAD non-wrapping group. These higher values of apical rotation resulted in augmented LV twist and LV torsion in LAD wrapping group compared with non-wrapping group (*P* < .001, < .001), respectively (Table 4, Figs. 5 and 6).

**Table 4:** Relationship of torsion and twist to clinical and echocardiographic variables

		Torsion	Twist			Torsion	Twist
**HR (b/min)**	R P	− .014 .905	.084 .487	mitral E/A	R P	− .015 .902	.123 .310
**SBP (mmHg)**	**R** **P**	− **.107** **.374**	− **.168** **.162**	**DT (ms)**	**R** **P**	− **.174** **.152**	− **.011** **.927**
**DBP (mmHg)**	**R** **P**	− **.183** **.126**	− **.119** **.323**	**E/E**_**a**_	**R** **P**	− **.083** **.493**	− **.147** **.225**
**LAD (mm)**	**R** **P**	− **.072** **.556**	− **.001** **.993**	**E**_**a**_**(cm/s)**	**R** **P**	− **.089** **.465**	**.080** **.510**
**LAV (ml/m**^**2**^**)**	**R** **P**	− **.101** **.405**	**.048** **.690**	**A**_**a**_**(cm/s)**	**R** **P**	− **.067** **.583**	**.180** **.137**
**ESD (mm)**	**R** **P**	− **.055** **.652**	− **.033** **.787**	**S**_**a**_**(cm/s)**	**R** **P**	**.105** **.385**	**.132** **.275**
**EDD (mm)**	**R** **P**	− **.090** **.460**	− **.048** **.694**	**ε**_**sys**_**% Global**	**R** **P**	**.070** **.563**	**.477** **.000**
**Septum (mm)**	**R** **P**	**.033** **.789**	− **.042** **.727**	**Mean TTP (ms)**	**R** **P**	− **.107** **.374**	− **.188** **.116**
**PW (mm)**	**R** **P**	− **.002** **.990**	**.001** **.993**	**TTP-SD (ms)**	**R** **P**	**.163** **.175**	− **.249** **.032**
**FS%**	**R** **P**	− **.064** **.600**	**.073** **.550**	**TTP-d (ms)**	**R** **P**	**.170** **.157**	− **.231** **.053**
**EF%**	**R** **P**	− **.063** **.605**	− **.065** **.591**	**Global SR**_**sys**_**(%)**	**R** **P**	**.007** **.952**	**.231** **.053**
**LVM (gm)**	**R** **P**	− **.132** **.278**	**.048** **.694**	**Global SR**_**e**_**(S**^**-1**^**)**	**R** **P**	− **.058** **.628**	**.553** **.000**
**LVMI (gm/m**^**2**^**)**	**R** **P**	− **.109** **.369**	**.111** **.362**	**Global SR**_**a**_**(S**^**-1**^**)**	**R** **P**	**.045** **.710**	**.078** **.520**
**Mitral E (cm/s)**	**R** **P**	− **.047** **.699**	− **.016** **.897**	**Basal Circ ε**_**sys**_**%**	**R** **P**	**.074** **.541**	**.546** **.000**
**Mitral A (cm/s)**	**R** **P**	**.016** **.896**	− **.090** **.458**	**Apical Circ SR**_**sys**_	**R** **P**	− **.027** **.820**	**.616** **.000**

*SBP* systolic blood pressure, *DBP* diastolic blood pressure, *LAD* left atrium dimension, *LAV* left atrium volume, *ESD* left ventricular end-systolic diameter, *EDD* left ventricular end-diastolic diameter, *EF* ejection fraction, *PW* posterior wall, *LVMI* left ventricular mass index, *E/A* ratio of early to late diastolic mitral inflow velocity, *DT* deceleration time, *E/Ea* ratio of early to early tissue doppler diastolic mitral flow, *E*_*a*_ early diastolic annular velocity, *A*_*a*_ late diastolic annular velocity, *S*_*a*_ peak systolic annular velocity, *ɛ*_*sys*_ peak systolic strain, *TTP* time to peak strain, *SD* standard deviation, *SR*_*sys*_ peak systolic strain rat, *SR*_*e*_ early diastolic strain rate, *SR*_*a*_ diastolic strain rate

### Relation of torsion and twist to clinical and echocardiographic variables

Pearson correlation coefficient analysis was used to test the relation of LV twist and all clinical and echocardiographic variables. No significant correlation was verified between torsion and twist and any of clinical and conventional echocardiographic parameters.

**Fig. 7:**
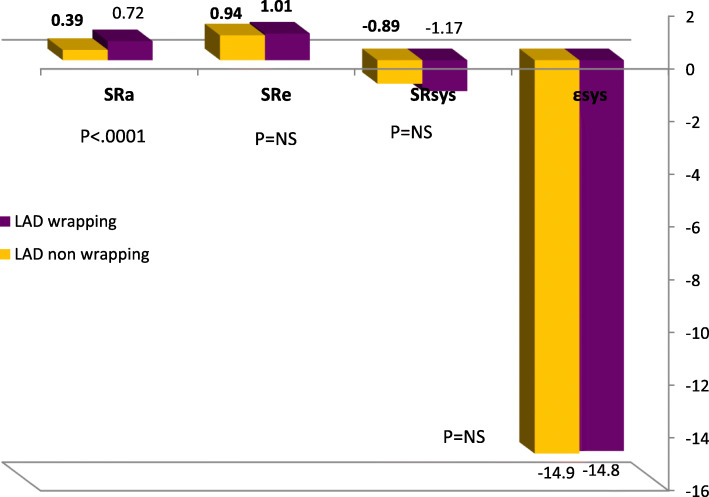
Longitudinal strain and strain rate in study groups

**Fig. 8:**
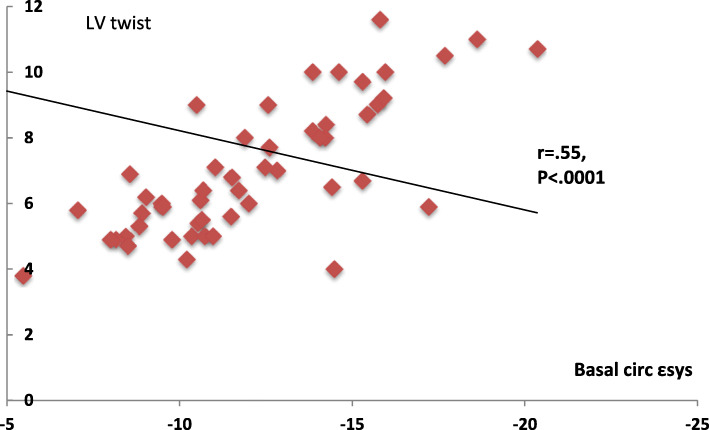
Correlation of LV twist to circumferential strain at the basal segment

On univariate analysis of the LV twist and LV longitudinal deformation (Fig. 7), LV twist showed a strong direct correlation to LV global **ε**_**sys**_ (*r* = .477, *P* < .000), modest inverse correlation to LV dyssynchrony as estimated by TTP-SD (*r* = − .25, *P* < .03, electromechanical delay as measured by TTP-d (*r* = − .23, *P* < .03), and direct correlation to LV systolic SR (SR_sys_) (*r* = .23, *P* < .05). Furthermore, the LV twist showed a strong direct correlation to diastolic function during early diastole as estimated by SR_e_ (*r* = .55, *P* < .000). Considering circumferential strain, LV twist showed a strong direct correlation to both basal (*r* = .56, *P* < .000) and apical rotation degree (*r* = .62, *P* < .000) (Fig. 8). No significant correlation was detected between LV torsion and any of the deformation variables.

Variables that were statistically significant in univariate analysis were introduced in a forward logistic regression model to estimate the independent predictors of LAD wrapping; these include hypertension diabetes mellitus, smoking, LVMI, DT, longitudinal SR_a_, circumferential ε_sys_ circ SR_sys_, circ SR_e_, circ SR_a_, apical rotation, and LV twist to discriminate LAD wrapping from LAD non-wrapping. From all these variables, LVMI [odds ratio .922, CI .860–.990, *P* < .02], DT [odds ratio .932, CI .877–991, *P* < .02], global longitudinal SR_a_ [odds ratio .000 CI .000–.271, *P* < .03], and LV apical rotation [.931, CI .876–.960 and *P* < .02] were independent predictors for LAD wrapping around LV apex in angiographically normal coronary arteries.

## Discussion

In the current study, we demonstrated that in patients with angiographically normal coronary arteries, long LAD that wrapped around LV apex ensured better longitudinal relaxation during late diastole, higher circumferential deformation at the LV apex, and augmented apical rotational mechanics. This deformational changes were resulting in higher LV twist despite no difference in LV ejection fraction measured by conventional methods. Longitudinal deformation in late diastole and LV apical rotation are independent predictors of LAD wrapping around LV apex in the presence of normal coronary circulation.

The present study is the first study, up to our knowledge, that investigated the relationship between LAD coronary artery anatomical features and cardiac mechanics in patients with angiographically normal coronary arteries.

Evaluation of LV function using 2D Echo-Doppler primarily is unable to describe detailed information about cardiac mechanics, specifically when there are no coronary arterial or structural heart diseases. Strain imaging using VVI is a semi-automated technique that is more precise and accurate in characterizing LV function than the conventional methods.

Additionally, as distinct from previous studies, in our study, measurements of longitudinal and circumferential strain incorporated specifically the mid myocardial layer which reflecting the true regional function allowing an early detection of subtle myocardial abnormalities [18, 19].

Lately published researches verified the crucial value of global longitudinal strain (GLS) as an important functional parameter that provides imperative diagnostic and prognostic information in patients presented with acute coronary syndromes [21, 22], and it has also been demonstrated as an independent predictor of significant CAD in patients with chronic stable angina [23]. Liou et al. [24] stated that GLS might be an initial marker of CAD in symptomatic patients. Furthermore, Stankovic et al. [25] claimed that 2D strain imaging was an exceptional choice to detect LAD stenosis especially in an acute setting. In parallel with these studies, the present study verified increased circumferential strain during diastole can predict the length of LAD and its wrapping around the LV apex in absence of LAD stenosis.

Kobayashi et al. [9, 10] investigated the association between anatomic features of LAD and patient’s outcome when presented with anterior ST-segment elevation myocardial infarction. They studied patients presented to CCU within 12 h after the development of suggestive symptoms of STEMI and subjected to primary percutaneous coronary intervention. Patients with LAD lesions confirmed to be the culprit lesions were divided into two groups, according to LAD anatomical features, as group 1: LAD wrapping (*n* = 871) and group 2: LAD non-wrapping (*n* = 224). They demonstrated that heart failure, significant arrhythmias, and LV mural thrombi were more common in the wrap-around LAD group. Additionally, LV ejection fraction was worse in the wrap-around LAD group (54.5% versus 58.7%, *P* < 0.006). Thereafter 3 years of follow-up, major adverse cardiac events (death, stroke/stent thrombosis) were higher (12.7% versus 5.4%, *P* < 0.002); death (6.6% versus 3.2%, *P* < 0.05), stroke (1.9% versus 0.5%, *P* < 0.12), stent thrombosis (5.6% vs 2.3%, *P* = 0.047), and severe heart failure (4.5% versus 1.4%, *P* < 0.03) were more prevalent in wrap-around LAD versus non-wrap-around LAD. Multivariate regression analysis revealed that LAD wrapping independently and considerably anticipated the occurrence of adverse cardiovascular events and severe heart failure in patients with an anterior STEMI.

In the current study, we perceived the explanation of different patient outcomes when LAD obstruction, with wrap-around apex, coexist as there is more loss of augmented function especially at LV apex; the LAD wrapping group had better mechanics and higher longitudinal relaxation. In addition, the main difference between wrapping and non-wrapping LAD groups is the higher circumferential function and augmented apical rotation that result in increased LV twist. So, not only a larger area of myocardium becomes vulnerable but a significant mechanical dysfunction is added when LAD obstruction supervenes, if LAD wraps around the apex.

Patients with proximal LAD occlusion are recognized by cardiac surgeons and cardiologists as a high-risk group with increased morbidity and mortality. However, not all proximal LAD lesions are of the same clinical relevance. Forty percent of LV myocardium is supplied by LAD, including the anterior wall and the anterior segment of the interventricular septum [26]. The LAD supplies all of this area, and much more when it wraps around the apex [3–5]. Therefore, according to our study, it is easy to understand now why proximal LAD disease can be a high-risk lesion. If LAD wrapping around the apex undergoes severe obstruction, not only a larger area of jeopardized myocardium is a consequence but also a loss of augmented LV function especially the apical rotational mechanics and the resulting LV twist.

During systole, the LV undergoes a unique twist motion with a counterclockwise rotation at the apex and a clockwise rotation at the base. The resulting twist during peak systole is 7.7 ± 3.5° [27]. This is followed immediately by rapid untwist at the end of the systole. This unique LV twisting is considered to be an important playing role not only in systolic but also in the diastolic function. LV twist generates positive torsional deformation forces that develop in the subepicardium layer that can be added to the opposing negative torsional motion originating in the subendocardium [27]. Eventually, the torque in the subepicardium impacts subendocardial deformation and the whole wringing motion of the heart will be affected with the reduction of rotational mechanics.

In our study, LV apical rotation and overall LV twist are much lower in LAD non-wrapping, and the larger part of LV twist is generated from apical counterclockwise rotation, so the peak LV twist is predominantly reduced in apical myocardial infarction; this might explain the reduction of LV torsion and even the occurrence of an apical aneurysm in apical myocardial infarction [28, 29].

Yet, in the present study, longitudinal strain did not differ between the study groups, and the longitudinal strain caused by the contraction of myocardial tissue in the endocardial layer and has a high sensitivity to myocardial ischemia which does not exist in our patients with angiographically normal coronaries. Several studies demonstrated that global longitudinal strain is a more sensitive and robust parameter for evaluating myocardial function compared to circumferential and radial strain [30, 31], while in normal myocardium, the circumferential strain is better reflecting both systolic and diastolic function [30, 31].

In addition, our study demonstrated that the left ventricular diastolic function as measured by deceleration time, longitudinal and circumferential strain, and strain rate during diastole (SR_a_ and SR_e_) was enhanced in a patient with long LAD that wraps around the apex. The underlying mechanism was reported in Wang et al.’s research [32]. Myocardial perfusion occurs mostly in diastole because systolic contraction transiently prevents coronary blood flow, especially to the subendocardium. So vascular turgor and large blood supply especially, to cardiac apex, will better perfuse the normal myocardium and predominantly augment the diastolic function. This diastolic function is initially affected if coronary artery stenosis develops.

Our study is the first to address the relationship between LAD length and LV function. We found that long LADs (i.e., those in which the vessel wraps around the apex) had higher values of LV function especially during diastole. From previous studies, LAD has a major impact on prognosis in patients undergoing primary PCI. This is probably as a result of a greater amount of the muscle supplied by this type of LAD, and therefore, a greater amount of myocardial necrosis when the vessel totally occludes [9, 10]. Furthermore, the augmented LV function associated with LAD will be regained after revascularization. This claim is further strengthened in previous studies when no differences were found between the LAD wrapping and LAD non-wrapping groups with regard to the presence and extent of collaterals and the blush grade (reflecting myocardial perfusion) in patients after LAD revascularization, and LAD length was the main determinant after intervention [8, 33].

Notably, our study showed a strong positive correlation between the LV twist and longitudinal and circumferential strain and strain rate especially at LV apex, and this explains the augmented LV twist in the LAD wrapping group was associated with better circumferential strain values compared to the LAD non-wrapping group. We could expect that proximal LAD stenosis in such a group with LAD wrapping to further deteriorate LV function to a greater extent and recommend utilization of longitudinal and circumferential strain as an early marker of LAD stenosis that better reflect LV dysfunction than LV EF%. This is in agreement with Stokke et al. [34] who reported a study showing strain imaging probably better reflects systolic function in patients with a preserved estimated LVEF.

There are numerous factors such as age, sex, diabetes mellitus, and hypertension that can affect cardiac mechanics in our study population, as examined by longitudinal and circumferential strain. Tadic et al. [35] demonstrated that patients with non-complicated diabetes and hypertension also had impaired LV longitudinal strain. In the present study, the prevalence of hypertension was higher in the LAD wrapping group while diabetes mellitus and smoking were more prevalent in LAD non-wrapping group which makes both groups at equal risk. Moreover, the ratio of male to female patients was similar in the study population, so the role of these risk factors on cardiac mechanics cannot be used to explain the difference in cardiac mechanics.

We included a real-life patient’s population with a variable risk profile who was referred to the catheterization laboratory for coronary angiography. However, we thought that the impact of these risk factors on cardiac mechanics might be equivalent. In the LAD wrapping group, hypertension was prevalent in 60% while diabetes mellitus and smoking were prevalent in 100% and 46% in LAD non-wrapping which represents a balanced exposure to cardiovascular risk. Compensatory left ventricular hypertrophy was existent in only 35% of the LAD wrapping group which renders their LVMI 115 g/m^2^ close to the normal values. Moreover, previous investigators reported that the longitudinal LV strain is decreased in patients with hypertension [13, 36] whereas our study group showed no significant difference in longitudinal strain, between LAD wrapped and non-wrapped patients which preclude the influence of hypertensive left ventricular hypertrophy on cardiac mechanics that characterize LAD wrapping group.

## Limitations

The present study has several limitations. First, it is a small sample-sized study with a limited number of patients; however, it can be explained by the strict exclusion criteria and single-center study. Second, image acquisition and analysis depend on many factors including image quality, operator experience especially with strain analysis software, and vendor variability. Third, concurrent risk factors like diabetes and hypertension might influence data results, despite invasive detection of LAD anatomical features in a healthy individual may be challenging. Finally, long-term follow-up could be performed to obtain clinical and prognostic figures especially when LAD obstruction occurs. Thus, large scale, multicenter, prospective, trials with enough power to examine the effect of all confounding factors are required to overcome these limitations.

## Conclusion

LAD wrapping around LV apex is a characteristic anatomical feature of coronary circulation that provides better myocardial relaxation, augmented circumferential, and rotational mechanics contrast to non-wrapping feature, in patients with normal coronary angiography. This could explain the poor prognosis and worse outcome in such population when acute LAD occlusion supervenes.

## Data Availability

The dataset supporting the results and conclusions of this article will be available from the corresponding author on request.

## Abbreviation

2D: Two-dimensional
CAD: Coronary artery disease
Circ: Circumferential
GLS: Global longitudinal strain
LAD: Left anterior descending artery
LV: Left ventricular
SR_a_: Atrial diastolic strain rate
SR_e_: Early diastolic strain rate
SR_sys_: Systolic strain rate
STEMI: ST-segment elevation myocardial infarction
TTP-d: Electromechanical delay
TTP-SD: Standard deviation of time to peak
VVI: Vector velocity imaging
ε_sys_: Peak systolic strain
